# Mutations Detected in Real World Clinical Sequencing during BTK Inhibitor Treatment in CLL

**DOI:** 10.21203/rs.3.rs-3837426/v1

**Published:** 2024-01-16

**Authors:** Jennifer Brown, Kiyomi Mashima, Stacey Fernandes, Aishath Naeem, Samantha Shupe, Rayan Fardoun, Matthew Davids

**Affiliations:** Dana-Farber Cancer Institute; Dana-Farber Cancer Institute; Dana-Farber Cancer Institute; Dana-Farber Cancer Institute; Dana-Farber Cancer Institute; Dana-Farber Cancer Institute; Dana-Farber Cancer Institute

## Abstract

We retrospectively analyzed 609 chronic lymphocytic leukemia (CLL) patients treated with BTK inhibitors (BTKis) at Dana-Farber Cancer Institute from 2014 to 2022. Among them, 85 underwent next-generation sequencing (NGS) during or after BTKi therapy (ibrutinib, 64; acalabrutinib, 13; pirtobrutinib, 7; vecabrutinib, 1). Patients with NGS at progression (N=36, PD group) showed more 17p deletion, complex karyotype, and previous treatments including BTKi, compared to ongoing responders (N=49, NP group). 216 variants were found in 57 genes across both groups, with more variants in the PD group (158 variants, 70.3% pathogenic, P<0.001). The PD group had a higher incidence of pathogenic variants (70.3%, P<0.001), including 32 *BTK*(*BTK* C481S/F/R/Y, L528W, and T474I/L) and 4 *PLCG2*mutations. Notably, a high VAF L528W mutation was found in a first line ibrutinib-resistant patient. *TP53, SF3B1*, and *NOTCH2*mutations were also significantly more prevalent in the PD group (P<0.01, P<0.05, P<0.05). Additionally, *MAPK* pathway gene mutations trended more common and had higher VAFs in the PD group (P=0.041). T474 mutations were found in 4 of 6 patients progressing on pirtobrutinib, and *BTK* L528W mutation can arise with both covalent and non-covalent BTKi therapy. These results also suggest that *RAS/RAF/MAPK* pathway mutations may contribute to BTKi resistance.

## Introduction

Bruton tyrosine kinase inhibitors (BTKi) targeting B cell receptor (BCR) signaling have dramatically improved the prognosis of chronic lymphocytic leukemia (CLL) in the last few years; however, acquired BTKi resistance remains a major problem.

*BTK* and downstream *PLCG2* mutations are the most commonly identified causes of BTKi resistance, usually at the cysteine 481 residue resulting in the replacement of the binding site of covalent BTKis, including ibrutinib (IBR) or acalabrutinib (ACA) [1, 2]. Novel non-covalent BTKi, such as pirtobrutinib, have been developed to overcome this resistance by binding regardless of the C481 residue [3]. We and others recently reported that mutations in the kinase domain of BTK (T474, L528) confer resistance to non-covalent BTKi [4, 5]. Preliminary results of a larger study have also demonstrated that mutations at the T474 gatekeeper residue and the L528W kinase impairing residue are associated with pirtobrutinib resistance [6].

Previous studies have also demonstrated that *BTK* independent mechanisms such as gain of 2p (*XPO1*), loss of chromosome 8p, and mutations of *CARD11, TP53* and *SF3B1* have all been associated with BTKi resistance in CLL [7–9]. However, mutational information during BTKi treatment, especially with noncovalent BTKi, remains limited. Moreover, resistance mechanisms caused by events other than *BTK/PLCG2* mutations occur in one-third to one-half of patients and the pattern of such mutations is still unclear. Here, we report the results of clinical sequencing of CLL patients (pts) treated with BTKi including ibrutinib (IBR), acalabrutinib (ACA) and pirtobrutinib (PIR) at Dana-Farber Cancer Institute between 2014 and 2022.

## Methods

### Patient population and collection of clinical characteristics

We retrospectively reviewed 765 BTKi treatment periods of 609 CLL pts who were treated with BTKis at DFCI between 2014 and 2022, and a total of 301 pts with 470 clinical sequencing results were available (**Supplementary Figure S1**). We excluded 216 cases in which clinical sequencing was done before or at a time unrelated to BTKi treatment, and ended with a total of 133 sequencing results. We focused on the 85 pts who had available clinical sequencing from at least one peripheral blood or bone marrow sample during (80.0%) or at next office visit within 3 months after the period of BTKi treatment (20.0%; median 22.5 days, range 8–78 days). We excluded 15 results due to absence of treatment effect assessment or other reasons (one examined after development of acute myeloid leukemia, one received BTKi but for graft-versus-host-disease treatment), and a total of 118 sequencing results were enrolled in this study. Sequencing was done at physician discretion as part of routine clinical practice.

### Targeted DNA Clinical Sequencing

The sequencing results come from a comprehensive clinical grade 88-gene next generation sequencing panel called the Rapid Heme Panel (RHP) that was developed at the Brigham and Women’s Hospital and the methodology has been previously published [10]. It includes most oncogenes and tumor suppressor genes frequently mutated in hematologic malignancies and is designed for genetic analysis of somatic mutations with an average depth of 1500X and estimated sensitivity of 3%. Chromosomal copy number loss and gains (CNAs) were also evaluated using read count analysis of clinical sequencing data. The predicted pathogenicity of each variant was assigned using VarSome to determine the classification by the American College of Medical Genetics and Genomics (ACMG) standards. In the analysis of mutational evolution, we defined the clones as follows; new clone: newly detected clone; vanished clone: completely undetected clone; decreased clone: decreased with VAF change > 0.1; increased clone: increased with VAF change > 0.1; stable clone: VAF change ≤ 0.1. If multiple different variants were observed in the same gene, they were prioritized in the order of new, increased, stable, decreased and vanished. Our clinical sequencing panel did not include the *BTK* gene until November 2019. However, only 5 tests from prior to that date were included, and then only as baseline measurements prior to any BTK inhibitor treatment, for sequential monitoring after BTK inhibitor initiation.

### Statistics

Differences between the two populations were evaluated by Fisher’s exact test or the Chi-square test as appropriate. All statistical tests were performed with EZR (Saitama Medical Center, Jichi Medical University, Saitama, Japan) [11].

## Results

### Characteristics of the patients

Patient characteristics for the 85 patients are shown in **Table 1**. The majority were male (75.3%), with a median age at diagnosis of 53 [29–87], and a median age of 60 [39–91] at BTKi start. 68.2% had IGHV unmutated disease, and 29.4% had 17p deletion. Thirty-one (36.5%) were receiving BTKi as first line therapy, 19 (22.3%) as second line, and 35 (41.2%) after multiple other therapies. 73 patients (85.9%) had only one BTKi (ibrutinib (IBR), 64; acalabrutinib (ACA), 9). 12 pts had multiple BTKis, 8 with two drugs with IBR first followed by ACA (N = 3, 37.5%), vecabrutinib (VEC) (N = 1, 12.5%), and PIR (N = 4, 50.0%); and 4 with three or more drugs (four had multiple BTKis prior to the current one: spebrutinib (SPE)-IBR now on ACA, and 3 with IBR-ACA, ACA-VEC or SPE-IBR-VEC now on PIR). The total number of patients in this cohort who received pirtobrutinib was 7. The median follow-up time for the BTKi treatment period was 87.9 months [21.0–104.9 months].

The clinical sequencing test was performed at disease progression in 36 pts (Group PD), with a median observation time from initiation of BTKi of 34.7 months, while 49 were not progressing, with a median time on BTKi of 26.3 months (Group NP). The testing done in NP patients included mutation screening during BTKi treatment for evaluation of an elevated peripheral lymphocyte count and/or residual lymph nodes not meeting PD criteria. In two cases with two or more sequencing results performed during both the NP and PD periods, only the NGS results obtained during the PD period were allocated to the PD group. Comparing the clinical features of the PD and NP groups (**Table 1**), gender and age at diagnosis are not significantly different (NP vs PD: male gender 79.6% vs 69.4% P = 0.317, median age at diagnosis: 53 [29–87] vs 52.5 [35–76], P = 0.971). In the NP group, 81.6% of the pts were treated with IBR and 18.4% with ACA. In the PD group, pts were receiving IBR in 68.6%, ACA in 11.4%, and PIR in 17.1%. In addition, the PD group was enriched for pts who were heavily treated and had prior history of BTKi, and both were significantly different compared to the NP group (NP vs PD, 1/2/3 + lines: 53.1%/28.6%/18.4% vs 13.9%/13.9%/72.2%, P< 0.001; prior BTKi history: 4.1% vs 27.7%, P = 0.0019), reflecting refractoriness to both overall treatment and BTKi.

**Figure 1:**
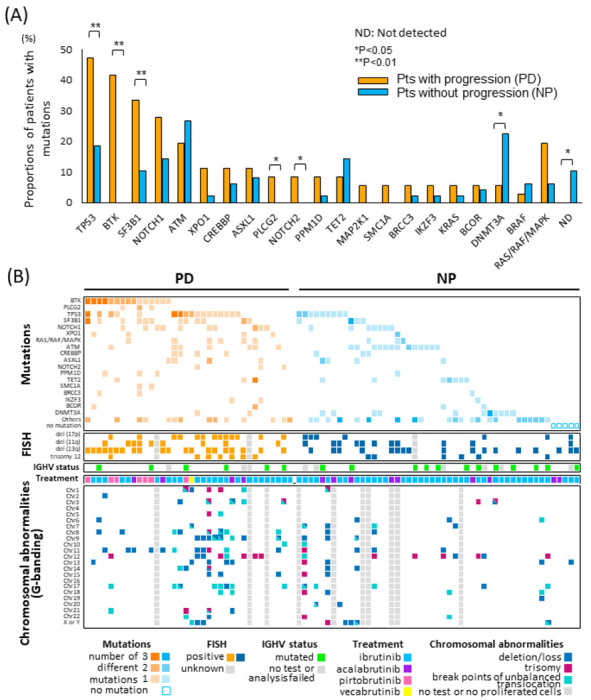
Major mutations and FISH and karyotype abnormalities detected in CLL patients with or without progression on BTKis The sequencing results come from a comprehensive clinical grade 88-gene next generation sequencing panel. The proportion of patients with mutations (1A) in PD and NP groups, and co-mutations and karyotype abnormalities from both FISH (17p deletion, 11q deletion, 13q deletion, and trisomy 12) and G-banding analysis (deletion or loss, trisomy, and breakpoints of unbalanced translocations) are analyzed (1B). One and two asterisks represent p values below .05 and .01, respectively.

28 of 36 pts in the PD group and 36 of 49 in the NP group were screened with both peripheral FISH status and karyotype at the same time (or within three months) of clinical NGS. At the closest time point to the clinical NGS, the proportion of TP53 mutated and del(17p) positive pts, but not del(11q) pts, was also significantly different between NP and PD groups, consistent with a previous report [12]. Pts with complex karyotype defined as either 3 + or 5 + abnormalities were significantly enriched in the PD group with higher prevalence of unbalanced translocations (NP vs PD, 3 or more abnormalities, 24.5% vs 55.6%, P = 0.0035; 5 or more abnormalities, 16.3% vs 41.7%, P = 0.0094; unbalanced translocations, 21.4% vs 42.4%, P = 0.0503). Among the pts with unbalanced translocations, the majority of the breakpoints were located at chromosomes 8 and 17 (38.9% and 33.3%, respectively), while chromosome 17 was the only recurrent breakpoint seen in more than 3 cases in the NP group (27.3%) (Fig. 1B, **Supp tableS1**). The unbalanced translocations at chromosome 8 caused 8p loss and/or 8q gains, which have been reported to be associated with BTKi resistance and refractory CLL [13–15].

### Overview of Sequencing Results

A total of 279 mutations were identified across our entire cohort, which included recurrent mutations (**Table 1**, Fig. 1B). Of these, 216 unique variants were found across 57 genes. The PD patients tended to have a higher number of mutations compared to those in the NP group. The median number of mutations per patient was 3 (ranging from 1 to 11) in the PD group and 2 (ranging from 0 to 8) in the NP group (P = 0.072). A larger overall number of variants and more pathogenic variants were identified in the PD group, with a total of 158 variants in 36 pts (56.6%), with 70.3% considered pathogenic, as compared to only 121 variants in 45 pts (43.4%) in the NP group, with 48.0% considered pathogenic (P < 0.001). Cases with recurrent copy number alterations (CNAs) by NGS were present only in the PD group (loss of 17p, 11q, 3p, and gain of 8q, 4q, 9p, 10q). Moreover, all patients with two or more CNAs had complex karyotype by G-banding at the same time points, and the PD group had a significantly higher number of karyotypic abnormalities.

#### BTK and PLCG2 mutations

Mutations identified in *BTK* included C481S (c.1442G > C) (10 pts), C481S (c.1441T > A) (6 pts), C481S (c.1442_1443delGCinsCT) (1 pt), C481R (3 pts), C481Y (2pts), C481F (1 pt), L528W (3pts), T474I (3pts) and T474L (2pts). Mutations in PLCG2 included L845F (2pts), D993H (1pt), D993Y (1pt) and R665W (1pt). With a median 30.4 months [1.0–117.0 months] on BTK inhibitor treatment, *BTK* mutations were detected only in the PD group (PD vs NP 41.7% vs 0%, P < 0.01), and at a rate lower than previously reported [1] (Fig. 1A). In the 15 cases with *BTK* mutations in the PD group, 7 of them progressed during IBR treatment, 2 during ACA, and 6 during PIR. None of the seven IBR treated pts had a prior history of BTK inhibitor treatment while one of the two ACA pts had prior IBR and SPE, and all of the PIR treated pts had a prior history of BTK inhibitors (3 IBR, 1 IBR and ACA, 1 IBR and VEC, 1 ACA and VEC). In the seven IBR treated pts, *BTK* C481S was detected in all of them along with the other C481 mutations C481R and C481Y in two and one pts, respectively. L528W was detected in a total of 2 cases on IBR with variant allele frequency (VAF) 2.1% and 62.2% (**Table 2**). Interestingly, this high VAF L528W mutation with C481S (2.1%) and C481R (10.2%) was detected during first-line IBR treatment. Two ACA treated pts only had C481S mutations; one had only one C481S mutation (c. 1442 G > C), and the other had two (c. 1442 G > C and c.1441T > A). In six PIR treated pts, four had *BTK* T474 mutations (two T474I alone, one T474I with C481S, one T474I with T474L). One of the other two PIR pts had L528W (20.4%) with multiple C481 mutations (C481S(c.1441T > A), 18.0%; C481S(c.1442_1443delGCinsCT), 4.0%; C481R, 16.4%; C481F, 4.0%; C481Y, 3.5%), while the other had C481S alone (C481S(c.1442G > C), VAF 5.6%).

A total of 5 *PLCG2* mutations were detected in three PD cases (R665W with VAF 1.6% during IBR; L845F with VAF 5.3% during PIR; D993H with VAF 7.7%, D993Y with VAF 3.1% and D1144G with VAF 0.3% during PIR, respectively). All three cases also had BTK mutations. No *PLCG2* mutation was detected in the NP group.

#### Distribution of mutations other than BTK and PLCG2

All the pts without any mutation in the RHP panel were in the NP group (10%, N = 5) and this difference was significant (NP vs PD, 0% vs 10.2%, P = 0.048). As described in Fig. 1A, *TP53* mutations were significantly enriched in the PD population (NP vs PD, 18.3% vs 47.2%, mVAF 12.8% vs 14.1%, P < 0.01). In addition, the proportion of pts with *SF3B1* and *NOTCH2* mutations was significantly higher in the PD group (P < 0.01, P<0.05). *NOTCH1* and *SMC1A* mutations were increased in the PD group but not significantly, possibly due to the small number of cases. Among four pts who developed Richter’s transformation during BTKi treatment (all IBR), two of them had *NOTCH1* mutation. In our cohort, *XPO1* mutations (four E571K and one D624G) showed a trend to enrichment in the PD group in the proportion of pts (NP vs PD 2.0% vs 11.1%, P = 0.079), with a significantly higher VAF (median VAF, NP vs PD 6.0% vs 44.4%, P = 0.021). *ATM* mutations have been reported to accumulate especially in CLL among lymphoid malignancies [16]. The total distribution of *ATM* mutations was not different between groups (NP vs PD, 13 of 50 vs 7 of 35, P = 0.52) nor was 11q deletion distributed differently. In contrast to the mutations above, the number of cases with *DNMT3A* mutations was significantly higher in the NP group (NP 12 mutations in 11 cases (22.4%), vs PD 2 mutations in 2 cases (5.56%), P < 0.05; median VAF, 1.95% vs 6.45%, P = 0.93). The VAF of *DNMT3A* mutations was very low in both the PD and NP groups (NP vs PD mVAF 2.0% vs 6.45%), so it is possible that these are myeloid CHIP mutations.

No other individual gene was significantly differently distributed, but when we consider an entire pathway, total mutations in *MAPK* related genes, *BRAF, NRAS, KRAS*, and *MAP2K1*, in the PD group were increased, and had higher VAFs, compared to the NP group (PD vs NP proportion of pts, 19.4% vs 6.1%, P = 0.0596, median VAF 32.0% vs 7.5%, P = 0.041). All the mutations among the 7 pts in the PD group (*BRAF* D594G; *MAP2K1* E203V and Q56P; *KRAS* A59G and K117N; and *NRAS* Q61H) have been previously reported as activating mutations [17–21]. This finding may indicate that *MAPK/ERK* activation can compensate for inhibited BCR signaling in driving cell survival and proliferation, as suggested by our prior work with PI3K inhibitor resistance [22]. These patients all had only one sampling time point, so it is unknown whether these mutations were pre-existing or acquired.

Analysis of co-mutation and karyotype abnormalities shows that 29 individuals in the NP group and only 3 patients with PD exhibited the absence of all of: *BTK, PLCG2, TP53, SF3B1* and *NOTCH1* mutations or 17p deletion; this difference was statistically significant (NP vs PD, 59.2% vs 8.33%, P < 0.001) (Fig. 1B). Among the 3 pts in the PD group without well-known resistance mutations and/or 17p deletion, one had a highly complex karyotype including 11q deletion and 8q gain. The other 2 pts had normal karyotypes, and one had *XPO1* (E571K, 45.0%) with *CSF3R* (M696T, 50.8%) mutations, the other had *EGFR* (P848L 42.5%) with *SMC3*(V1087I 52.9%).

Four cases in the PD group progressed with Richter’s transformation during BTKi treatment and all of them had pathogenic mutations and/or CNAs. *TP53* mutations were seen in three cases, *NOTCH1/2* mutations in two cases, *ASXL1* in two cases and *MAPK* related gene mutations (*KRAS, BRAF*) in two cases.

### Clonal Evolution During BTKi Treatment

**Figure 2:**
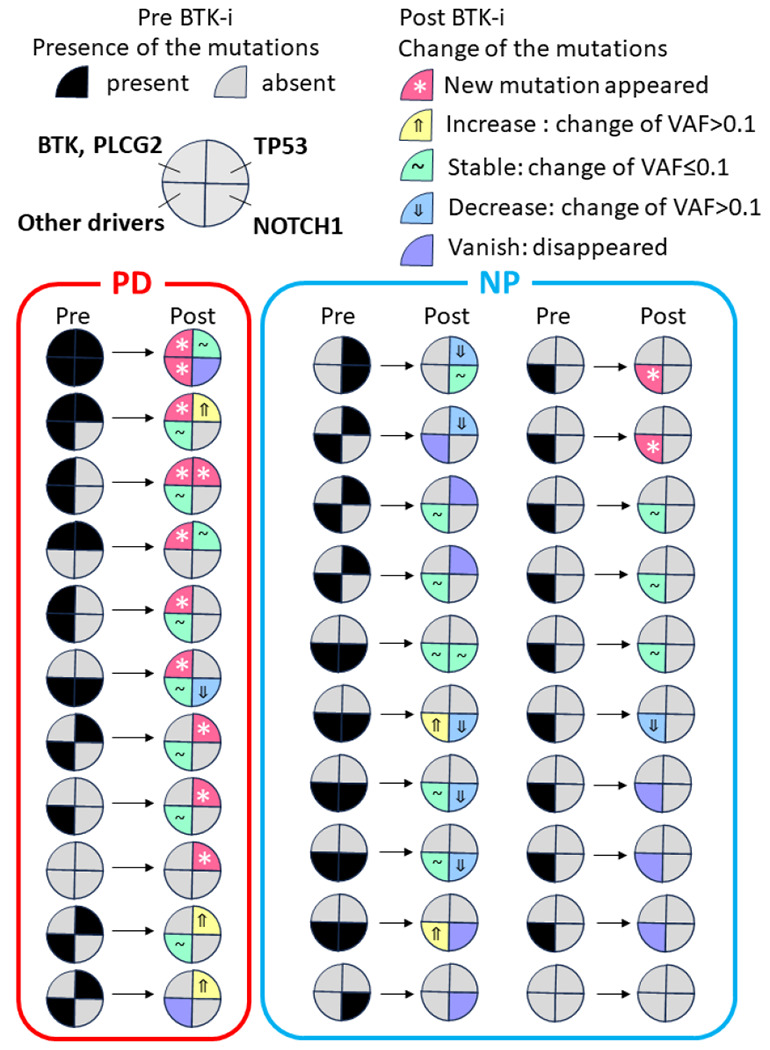
Comparison of clonal evolution of key mutations according to BTK response 11 in the PD group and 20 in the NP group had paired clinical sequencing results before and during BTKi treatment, and each circle represents one patient. Mutations were defined as follows: new clone: newly detected clone; vanished clone: completely undetected clone; decreased clone: decreased with VAF change >0.1; increased clone: increased with VAF change >0.1; stable clone: VAF change ≤ 0.1.

Of the 85 patients in our cohort, 11 in the PD group and 20 in the NP group had paired clinical sequencing results before and during BTKi treatment. A total of 46 mutations were newly emerged (PD vs NP, 26 vs 20) and 7 showed increasing VAF (PD vs NP, 4 vs 3) including non-CLL drivers. Importantly, all patients in the PD group with serial samples exhibited at least one increased and/or newly emerged *BTK* or *TP53* mutation during BTKi treatment (Fig. 2). The proportion of patients with evolving or new mutations of CLL driver genes during treatment was significantly higher in the PD group (100% in PD vs. 2% in NP, P < 0.001), which is consistent with a recent report [23]. In the PD group, only three patients showed the decrease or disappearance of a CLL driver mutation during BTKi treatment. In contrast, in the NP group, two *TP53* mutations, six *NOTCH1* mutations, and four other driver mutations completely disappeared during BTKi treatment.

#### Evolution of BTK mutation clones during treatment

We further focused on the 6 of 14 cases with *BTK* mutations for whom paired sequencing was available (two patients had 3 results) both before and during or after BTK inhibitor treatment (Fig. 2). All of their second (or in one case third) timepoints were examined during PIR treatment. Their first sequencing results were taken during IBR treatment in four pts (three PD #1, #2, #3, one responding #4), and the other two were during VEC (Case #5) and venetoclax (Case #6). After only 8 months of PIR treatment, case #1 developed a new *BTK* L528W and multiple C481 mutations with disease progression. C481R reduced, but C481S(c.1442_1443delGCinsCT) increased and C481F, S (c.1441T > A), and Y newly appeared. This finding is potentially consistent with early observations by our group and others that suggest that non-C481S, or C481S together with other drivers, can still occur or increase during pirtobrutinib therapy [4, 6, 24]. Cases #2 and #3 developed T474I mutation after 15 and 24 months on PIR with 14.3% and 15.2% VAF, respectively. In case #4, disease progression on PIR was associated with three newly detected *PLCG2* mutations, D993H (VAF 7.7%), D993Y (3.1% VAF) and D1144G (0.3%), while several mutations present at disease progression on IBR diminished with PIR (*BTK* C481S (c.1442G > C) VAF 30.3–0.8%; *TP53* mutation E258D VAF 43–4.1% and *BTK* C481S (c.1442T > A) (VAF 2% to undetectable). Case #5 already had a small clone of *BTK* T474I that developed during ACA before PIR initiation, and this clone developed to high VAF of 89.8% during PIR, as we have previously reported [4]. This patient also acquired *BTK* T474L mutation at relapse on PIR. Case #6 had a large clone of T474I with VAF of 71.8% at progression after 16 months of PIR treatment, but this patient already had this clone (T474I VAF 71.8%) 5 months prior to progression, although it had not been seen before PIR treatment. Detailed analyses of cases 1 and 5 were already presented in our previous paper [4].

## Discussion

In this study, we retrospectively analyzed targeted NGS data in pts with CLL on BTKi treatment, including both covalent (IBR and ACA), and non-covalent (PIR and VEC) drugs, to evaluate further the resistance mechanisms to BTKis. We detected *BTK* (N = 32) or *PLCG2* (N = 4) mutations, including *BTK* C481S, C481F, C481R, C481Y, L528W, T474I, and T474L, in 41.7% of the PD group, along with 8.3% who had *PLCG2* mutations, while none of the NP group had either *BTK* or *PLCG2* mutations. In our cohort, all *PLCG2* mutations co-occurred with *BTK* mutations. The lower detection rate of BTK mutations in our cohort compared to initial studies [1, 15, 25] is nonetheless consistent with more recent studies [15, 26]. Of note, a L528W mutation with high VAF (60.2%) was detected in this IBR resistant cohort, even though this mutation has been described to date mainly during PIR and zanubrutinib treatment [4, 27]. More data are needed on the distribution of non-C481 mutations in progressors on covalent BTKis.

Five T474 mutations were detected during both PIR and VEC treatments, and 4 of 6 (66.7%) PIR PD patients carried T474 mutations (T474I and T474L) as the dominant *BTK* mutations. These mutations were detected earlier with higher VAF at the PIR resistant point compared to the timing of C481S mutation during IBR progression (median detected time at progression: T474 14 months vs C481S 173 months). However, all of these patients treated with PIR had been previously treated with other BTKis and already carried resistance mutations, which likely played a major role in the more rapid development of resistance. The 3 pts with C481S mutations detected during PIR had the same mutations before PIR treatment, and the VAF decreased in all cases. Rapid selection and aggressive progression of T474 mutations were the main cause of resistance to PIR in our cohort. These results are overall consistent with the recently reported cohort from the BRUIN trial, albeit with fewer L528W mutations [6].

More than half of our PD cohort did not carry *BTK* or *PLCG2* mutations (58.3%, N = 21) at progression. Among those pts without *BTK* related mutations, 57.1% had *TP53* mutations, 47.6% had deletion 17p, 28.6% had *SF3B1*, and 28.6% had *NOTCH1* mutations. In our 10 PD and 20 NP cases with RHP data pre-BTKi treatment, all but one PD case showed new mutation or increased VAFs of at least one of *BTK/TP53/SF3B1*, while the NP cases with *TP53* or *SF3B1* mutations (N = 8) at their pre timepoint did not show increased VAF or new mutations (2 stable, 4 decreased, 2 vanished) during BTKi treatment. Previous reports have associated TP53 aberrancy with shorter progression free survival (PFS) and demonstrated increased VAF with progression during IBR treatment [15, 28, 29]. However, whether *TP53* aberrancy contributes directly to CLL resistance to BTKi is still controversial. In our cohort, the number of pts with *SF3B1* mutation was significantly enriched at progression, and the number of those with *NOTCH1* mutations also tended to increase in the PD group. A few reports have demonstrated a direct effect of *NOTCH1* activation on ibrutinib resistance, but the distribution and correlation of *NOTCH1* and *SF3B1* mutations in BTKi resistant cohorts remains controversial, and the mechanisms are not well described [15, 30, 31]. In addition, when we consider an entire pathway, mutations in *MAPK* related genes, *BRAF, NRAS, KRAS*, and *MAP2K1*, were enriched in the PD group and had significantly higher VAFs. Previous reports have demonstrated that CD79B overexpression activated MAPK leading to IBR resistance in ABC-diffuse large B cell lymphoma and *MAPK-ERK* inhibition was effective in IBR resistant mantle cell lymphoma [32, 33]. *MAPK* pathway activation could potentially bypass BTK inhibition and allow CLL cells to proliferate.

Recent studies showed that complex karyotype and unbalanced translocations are associated with adverse prognosis in CLL [34–36]. In our study, we detected 18 unbalanced translocations in 14 PD pts and 11 in 8 NP pts. Chromosome 17 was the only common recurrent breakpoint in both groups; however, chromosome 8 related translocation, the most frequent breakpoint in the PD group, was observed only once in the NP group. Among our 7 pts with derivative chromosome 8 in the PD group, recurrent breakpoints were 8p23 (N = 2, causing 8p loss), 8q11.2 (N = 2, causing 8q loss), and biallelic loss of whole 8p arms (N = 2, causing 8p loss). Previous studies showed that 8p loss, especially loss of TRAIL-R, was associated with ibrutinib resistant disease in CLL [13, 15, 37]. In addition, 8p carries several tumor suppressor genes, and its loss has been shown to be related to venetoclax resistance in CLL and adverse prognosis in solid tumors such as breast, liver and prostate cancers [38, 39]. Further studies are needed to clarify the mechanisms of resistance associated with 8p loss.

In summary, this retrospective cohort analysis reports the results of clinical sequencing during BTKi treatment for CLL. Beyond *BTK/PLCG2* and *TP53* mutations, our results suggest that *RAS/RAF/MAPK* pathway mutations are also related to BTKi resistance. Our results show that *BTK* L528W can occur during both covalent (IBR) and non-covalent (PIR) BTK inhibitor therapy and may be related to ibrutinib resistance. Four of 6 patients who progressed on PIR had T474 mutations in about a year. Continued study of the incidence and evolution of mutations in BTK L528, T474 and C481, during both covalent and non-covalent BTKi therapy are required as we try to optimize our therapeutic sequencing for maximal patient benefit.

## Figures and Tables

**Figure 3:**
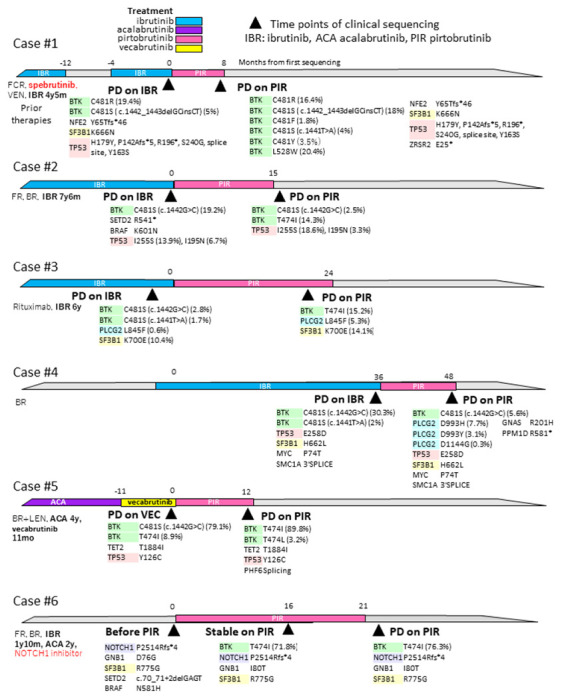
Evolution of mutation clones during treatment In 14 cases with BTK mutations, 6 had two (and two had three) paired sequencing results before and during/after BTKi treatment. Prior therapies are shown on the left side, and colored bars represent BTKi treatment periods. Black triangles represent the time points of NGS examination, and all mutations are described below each time point.
